# Fatal outcome of late-onset angiotensin-converting enzyme inhibitor induced angioedema: A case report

**DOI:** 10.1097/MD.0000000000011695

**Published:** 2018-08-03

**Authors:** Jone Jackeviciute, Vidas Pilvinis, Rugile Pilviniene

**Affiliations:** a Medical Academy; b Department of Intensive Care; c Institute of Physiology and Pharmacology, Medical Academy, Lithuanian University of Health Sciences, Kaunas, Lithuania.

**Keywords:** adverse reaction, angioedema, angiotensin-converting enzyme inhibitor, angiotensin-converting enzyme inhibitor side effects, upper airways

## Abstract

**Rationale:**

Angiotensin-converting enzyme (ACE) inhibitors are one of the most used medication among patients with arterial hypertension. In most cases, ACE inhibitors caused side effects are mild; however, from 0.1% to 0.7% of patients can develop life threatening adverse effect, angioedema. Unlike histamine mediated, ACE inhibitor-related angioedema can develop at any time during the treatment course.

**Patient concerns:**

An 89-year-old woman with a medical history for arterial hypertension, ischemic heart disease, heart failure, chronic atrial fibrillation developed ACE inhibitor-induced angioedema after 5 years of daily ramipril administration.

**Diagnoses:**

Arterial hypertension, ischemic heart disease, heart failure, chronic atrial fibrillation and late onset ACE inhibitor-induced angioedema.

**Interventions:**

The ACE inhibitor was used for arterial hypertension on a daily basis for the past 5 years. Patient developed airway obstruction requiring intubation. Standard therapy with epinephrine, methylprednisolone and clemastine was administered. Treatment was ineffective, considering that angioedema persisted.

**Outcomes:**

Angioedema resolved after 13 days from the discontinuation of ramipril. Death due to cardiopulmonary insufficiency occurred 24 days after the admission to intensive care unit, despite full clinical resolution of ACE inhibitor-induced angioedema.

**Lessons:**

Our case highlight the importance of educating clinicians about ACE inhibitor-induced angioedema, as potentially fatal adverse drug reaction. Considering the fact, that no laboratory or confirmatory test exist to diagnose ACE inhibitor-induced angioedema, clinicians’ knowledge is the key element in recognition of ACE inhibitor-related angioedema.

## 1 Introduction

Angiotensin-converting enzyme (ACE) inhibitors are one of the most commonly used medication among patients with arterial hypertension (AH), also the key medication for congestive heart failure and proteinuria in diabetic and nondiabetic nephropathy.^[1]^ For instance, ACE inhibitors are prescribed in 65% of patients with coronary artery disease and in 71% of patients with heart failure.^[2]^ Although ACE inhibitors are well established, medication side effects may present. From 0.1% to 0.7% of patients using ACE inhibitors can develop life-threatening adverse effect, angioedema, which is characterized as nonallergic, because it is not associated with degranulation of mast cells by immunoglobulin-E.^[3]^ Angioedema can present in different body locations, for example, face, lips, tongue, throat, and viscera.^[4,5]^ Upper respiratory tract involvement may lead to airway obstruction and acute respiratory distress if not recognized from the beginning.^[6]^

Moreover, the time of presentation of the angioedema in relation to ACE inhibitor therapy varies. Majority of angioedema cases were documented during first 30 days after ACE inhibitor exposure, although angioedema may develop at any time during the treatment course.^[7,8]^ Delayed angioedema may be associated with poor recognition, because identifying the association between initiation of the ACE inhibitor therapy and symptoms is difficult.^[9]^ We present a case of late-onset ACE inhibitor-induced angioedema, which resulted in cardiac arrest due to severe airway obstruction.

### 1.1 Ethics approval and consent to participate

Approval to analyze the case file was given by the patient.

### 1.2 Case presentation

An 89-year-old Caucasian female with a medical history for AH, ischemic heart disease (coronary artery bypass surgery without prolonged ventilation), heart failure, chronic atrial fibrillation presented to emergency department with dyspnea, difficulty in speaking, hoarseness of voice, and edema of the neck was presented in this study (chronological medical history is provided in Table 1). All of the symptoms occurred 2 days ago. Medical records revealed that coughing occurred about a month ago, and gradually became worse. Pneumonia was suspected; therefore, blood tests were collected and chest X-ray was performed. During X-ray, the patient developed airway obstruction requiring intubation. For further treatment, the patient was admitted to the intensive care unit (ICU). Blood tests revealed slight leukocytosis and increased C-reactive protein levels. Empiric antibiotic therapy was initiated. It was revealed from medical records that she was on the following medication: warfarin, metoprolol, amlodipine, torsemide, as well as ACE inhibitor (ramipril) on a daily basis for the past 5 years. No recent changes in medication or dose were performed; also, no history of smoking, seasonal or medication allergies, and no family history of angioedema were reported. After the patient was sedated, ventilated, and monitored for 24 hours in ICU, it was decided to wean her off the ventilator. Ability for spontaneous breathing (SB) was assessed with T-piece test (T). SBT was performed for 60 minutes and was well tolerated: no tachycardia, no tachypnea, and no signs of increased work of breathing presented. The patient was conscious and responsive, therefore extubated. However, 1 hour after extubation, desaturation and partial airway obstruction developed, consequently urgent reintubation was decided. Intubation presented as difficult, due to narrowing of trachea below the vocal cords. Bronchoscopy was performed to evaluate the unknown origin of trachea narrowing; however, bronchoscope could not pass through intubation tube. Further evaluation was performed with contrast-enhanced neck and chest computed tomography (CT) scan. The CT scan revealed soft tissue edema, which involved the base of the tongue and trachea from cricoid cartilage and up to 2.6 cm below. Standard therapy with epinephrine, methylprednisolone, and clemastine was given. However, treatment was highly ineffective because of persistent soft tissue swelling. All of previously used medications were discontinued, including ACE inhibitor. Four days after discontinuation of ACE inhibitor, a sudden drop in oxygen saturation (SpO_2_) presented, capnography revealed obstructive pattern; therefore, suction with catheter was initiated. Catheter could not pass through intubation tube, due to the obstruction. Manual ventilation was performed; however, oxygenation was inadequate and severe hypoxia resulted in cardiac arrest. Cardiopulmonary resuscitation (CPR) was started immediately after the patient became pulseless. During CPR, the patient received 14 mg of epinephrine and 250 mmol of bicarbonate. Spontaneous circulation returned after 38 minutes. Emergency bronchoscopy revealed blood clot and soft tissue edema fully obstructing the distal end of endotracheal tube. After blood clot was removed, air entry improved and SpO_2_ increased. Angioedema was suspected; therefore, for further identification of the underlying cause and differentiation of angioedema, diagnostic tests were performed. C3, C4, IgE levels, and C1 esterase inhibitor protein levels were in the normal range; therefore, hereditary angioedema was ruled out and ACE inhibitor induced angioedema diagnosed. Two units of fresh frozen plasma (FFP) were administered; however, no improvement was noted. Neurological examination was performed 24 hours after restoration of spontaneous circulation: eye opening response to pain, no verbal response, and no motor responses to painful stimuli (Glasgow Coma Scale – 4). Patients’ corneal reflex was intact and pupils reactive to light bilaterally. Head CT scan revealed signs of hypoxic ischemic brain damage. Angioedema resolved after 13 days from the discontinuation of ACE inhibitor. Patient did not regain consciousness, despite full clinical resolution of ACE inhibitor induced angioedema. Death due to cardiopulmonary insufficiency occurred 24 days after the admission to ICU.

**Table 1:**
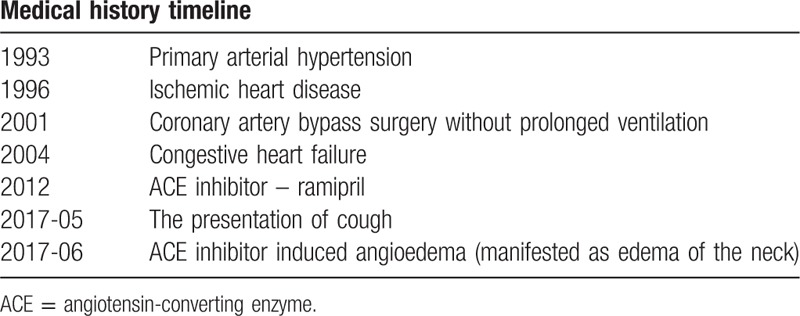
Medical history timeline.

## 2 Discussion

The key element to ensure adequate management of the patient and optimal treatment strategy is to determine whether histamine or bradykinin is involved into the angioedema development. ACE inhibitor induced angioedema is classified as bradykinin-mediated, because bradykinin interferes with the release of the main mediators of endothelial vasodilation function: nitric oxide, prostacyclin, and endothelium-derived hyperpolarizing factor.^[6,10]^ Differentiation between these 2 forms may be difficult, considering that no validated diagnostic test is available.^[6]^ Therefore, the main diagnostic tool involves exclusion of other types of angioedema, for example, a valuable marker for the differential diagnosis of hereditary angioedema over other types of angioedema is low serum C4 level.^[11]^ In our case, C4 level was in the normal range; as a result, hereditary angioedema was excluded. In addition, elimination of alternative pathologies manifesting as angioedema should be taken into consideration, for instance allergic reactions, facial cellulitis, acute contact dermatitis, photodermatitis, dermatomyositis, facial lymphedema, and superior vena cava syndrome.^[12]^ Furthermore, the cause of angioedema may be distinguished by assistance of medical history, current medication, family history, exposure to any known allergens, and timing of the angioedema presentation.^[13]^ Therefore, the ACE-inhibitor induced angioedema should be suspected for the patients who are using ACE-inhibitor; alternative pathologies were excluded and patients who are at a significantly increased risk for ACE-inhibitor caused angioedema development. The factors, which are associated with enhanced risk are age > 65 years, female gender, cough caused by ACE inhibitor, heart failure, black race, seasonal allergies, and history of smoking.^[14–16]^ In our case, the patient presented with some risk factors for angioedema: female gender, > 65 years, cough caused by ACE inhibitor, and heart failure. However, ACE-inhibitor caused angioedema can develop in patients without any known risk factors.^[17]^

Regardless of fatal ACE inhibitor related angioedema presentations, these conditions are frequently missed, misdiagnosed, or attributed to other causes.^[18]^ The main factor that complicates the diagnosis is the time from the beginning of ACE inhibitor therapy to the presentation of angioedema. Even though, about 50% of ACE inhibitor induced angioedema cases occur during the first week of ACE inhibitors use, it can occur years after initial treatment.^[19]^ In our case, angioedema presented after 5 years of daily ramipril usage. This delayed presentation is similar to the previous cases, which indicate ACE inhibitor daily administration for 7, 10, or even 23 years.^[17,20]^

The treatment of angioedema depends on severity of upper airway involvement, which can range from mild swelling to fatal airway obstruction.^[21,22]^ The most important component in all of the angioedema incidents is discontinuation of the medication, accompanied by airway protection. However, withdrawal of ACE-inhibitor is not always effective.^[6]^ ACE inhibitor caused angioedema tends to be persistent, considering that the average time from edema onset to full resolution takes about 30 hours, even though it can last up to 5 days.^[22,23]^ In our case, the complete resolution of angioedema presented after 13 days from the ramipril discontinuation. The longer resolution time is consistent with prior studies, which have shown that in severe cases, the interval between symptoms and angioedema resolution was approximately twice as long compared with mild forms.^[22,24]^ Moreover, standard treatment with antihistamines, corticosteroids, and epinephrine is ineffective due to ACE inhibitor induced angioedema pathophysiology. Although lack of response to regular treatment can be used as a clinical clue for differential diagnosis. Lack of response to regular treatment can be used as a clinical clue for differential diagnosis. Minimal improvement after administration of standard therapy has led to a search for substitute treatment. Considering that ACE inhibitor induced angioedema pathophysiologic mechanisms are similar to the hereditary angioedema, several novel therapies available for treatment of hereditary angioedema may be effective in ACE inhibitor induced angioedema management. Although this is promising, the increased efficacy must be weighed against the increased costs, for example, 1 dose of icatibant costs approximately about $10,000.^[25,26]^ FFP is one of the treatment options, because it has identical kininase II to ACE, and therefore leads to degradation of bradykinin.^[27]^ Optimal dosing strategy is not determined. However, published literature shows that early administration, immediately after onset of angioedema, may be required for success. Precise dose recommendation also cannot be made on the current literature; the patients receive from 1 to 4 of FFP units.^[26]^ In successful cases, significant remission occurs 2 to 4 hours after FFP administration.^[28]^ In our case, we did not receive a positive effect after FFP treatment; it may be due to severe form of angioedema, as well as postponed administration.

ACE inhibitor induced angioedema is recognized as a life-threatening condition; however, it is rarely fatal.^[29]^ Population-based study of angioedema-associated mortality showed that from 1999 to 2010, about 2000 cases of death were registered; however, only 18 cases were specifically due to ACE-inhibitors.^[30]^

## 3 Conclusion

Our case highlights the importance of educating clinicians and emergency specialists about ACE inhibitors induced angioedema, as a potentially fatal drug reaction. Considering the fact that no laboratory or confirmatory tests exist to diagnose ACE inhibitors induced angioedema, clinicians’ knowledge and experience is the key element in recognition of ACE inhibitors induced angioedema. Moreover, factors associated with increased angioedema presentation and delayed forms of angioedema should be taken into the consideration, as well as awareness of the pathophysiology, as it could ensure appropriate treatment and management of the condition, whenever standard therapy is not effective.

## Author contributions

**Conceptualization:** Jone Jackeviciute.

**Data curation:** Vidas Pilvinis, Rugile Pilviniene.

**Project administration:** Vidas Pilvinis, Rugile Pilviniene.

**Supervision:** Vidas Pilvinis, Rugile Pilviniene.

**Visualization:** Jone Jackeviciute, Vidas Pilvinis, Rugile Pilviniene.

**Writing – original draft:** Jone Jackeviciute, Vidas Pilvinis, Rugile Pilviniene.

**Writing – review & editing:** Jone Jackeviciute, Vidas Pilvinis, Rugile Pilviniene.
